# Draft Genome Sequence of *Vibrio parahaemolyticus* SNUVpS-1 Isolated from Korean Seafood

**DOI:** 10.1128/genomeA.00132-12

**Published:** 2013-02-07

**Authors:** Jin Woo Jun, Ji Hyung Kim, Casiano H. Choresca, Sang Phil Shin, Jee Eun Han, Se Chang Park

**Affiliations:** a Laboratory of Aquatic Biomedicine, College of Veterinary Medicine and Research, Institute for Veterinary Science, Seoul National University, Seoul, Republic of Korea; b Korea Institute of Ocean Science & Technology, Ansan, Republic of Korea

## Abstract

*Vibrio parahaemolyticus* is the leading cause of food-borne diseases, and several pathogenic strains cause global gastroenteritis outbreaks. Here, we report a draft genome sequence of *V. parahaemolyticus* SNUVpS-1, which was isolated from seafood in a fishery market in the Republic of Korea and contained TL, *toxR*, and *toxRS*^old^ genes. The current draft genome sequence will contribute to the effort to monitor the spread of *V. parahaemolyticus* seafood isolates and clinical isolates.

## GENOME ANNOUNCEMENT

*Vibrio parahaemolyticus* is a curved, rod-shaped, oxidase-positive, facultatively aerobic, non-spore-forming, Gram-negative, and motile bacterium. As one of the most important causes of gastroenteritis, *V. parahaemolyticus* is associated with the consumption of raw or improperly cooked contaminated seafood (1). *V. parahaemolyticus* is recognized globally as an important human pathogen (1–3). *V. parahaemolyticus* pandemic strains, such as O3:K6, contain a unique *toxRS* sequence that is associated with the current pandemic (4, 5). Increasingly, there have been reports of antibiotic resistance in *Vibrio* species. The emergence of microbial resistance to multiple drugs is a serious clinical problem that impedes treatment and increases fatality rates (4, 6). Due to the consumption of raw finfish and shellfish, East Asians, especially Koreans and Japanese, have an increased risk of gastroenteritis caused by *V. parahaemolyticus* infection (1).

To date, only the genome sequences of clinical isolates, such as O3:K6 *V. parahaemolyticus* strain RIMD2210633, O4:K12 strain 10329, O3:K6 strain AQ3810, O4:K68 strain AN-5034, and strain 16, have been reported and deposited in GenBank (7–9). In the present study, *V. parahaemolyticus* SNUVpS-1 was isolated from seafood (corb shell, *Cyclina sinensis*) from a fishery market in Seoul, Republic of Korea (4). It contained the thermolabile hemolysin gene (TL gene), the *V. parahaemolyticus toxR* gene, and the *toxRS* sequence of the O3:K6 clone isolated before 1995 (*toxRS*^old^) (4). In addition, it evidenced multiple-antibiotic resistance and was found to contain the antibiotic-resistance genes *tetA* and *strB* (4).

*V. parahaemolyticus* SNUVpS-1 genomic DNA was extracted as described previously (10) and was sequenced using standard shotgun sequencing methods using a 454 GS-FLX Titanium sequencing system (Roche) by Macrogen in the Republic of Korea. The sequence data consisted of 253,436 reads (mean length, 708.26 bp), providing 36-fold coverage. *De novo* assembly of the whole sequencing reads was performed with a Genome Sequencer (GS) *de novo* assembler (v.2.6). Sixty contigs, with a minimum length of 500 bp, were obtained. The draft genome of *V. parahaemolyticus* SNUVpS-1 was 5,241,845 bp in length with a G+C composition of 45.23%. A total of 4,705 open reading frames (ORFs) were discovered in the draft genome that was structured with 60 contigs.

In conclusion, the sequence data generated here will contribute to the understanding of genome variability and the epidemiology of *V. parahaemolyticus* seafood isolates, as well as of clinical isolates in future genomic studies.

### Nucleotide sequence accession number.

The draft genome sequence for *V. parahaemolyticus* SNUVpS-1 is available in GenBank under the accession no. AMRZ00000000.
